# Integrating metabolomics for precision nutrition in poultry: optimizing growth, feed efficiency, and health

**DOI:** 10.3389/fvets.2025.1594749

**Published:** 2025-05-22

**Authors:** Mohamed E. Abd El-Hack, Ahmed A. Allam, Ahmed K. Aldhalmi, Mahmoud Kamal, Muhammad Arif, Abdullah S. Alawam, Hassan A. Rudayni, Ayman E. Taha, Ayman A. Swelum, Ahmed A. Elolimy, Mahmoud Madkour, Elwy A. Ashour

**Affiliations:** ^1^Department of Industrial Pharmacy, College of Pharmaceutical Sciences and Drug Manufacturing, Misr University for Science and Technology (MUST), Giza, Egypt; ^2^Department of Biology, College of Science, Imam Mohammad Ibn Saud Islamic University (IMSIU), Riyadh, Saudi Arabia; ^3^College of Pharmacy, Al-Mustaqbal University, Babylon, Iraq; ^4^Animal Production Research Institute, Agricultural Research Center, Dokki, Giza, Egypt; ^5^Department of Animal Sciences, College of Agriculture, University of Sargodha, Sargodha, Pakistan; ^6^Animal Husbandry and Animal Wealth Development Department, Faculty of Veterinary Medicine, Alexandria University, Alexandria, Egypt; ^7^Department of Animal Production, College of Food and Agriculture Sciences, King Saud University, Riyadh, Saudi Arabia; ^8^Department of Integrative Agriculture, College of Agriculture and Veterinary Medicine, United Arab Emirates University, Al Ain, United Arab Emirates; ^9^Animal Production Department, National Research Centre, Dokki, Giza, Egypt; ^10^Poultry Department, Faculty of Agriculture, Zagazig University, Zagazig, Egypt

**Keywords:** metabolomics, growth, feed efficiency, poultry, precision nutrition

## Abstract

Nutrition is an important factor in poultry production. This review highlights how precision nutrition improves poultry performance through metabolomics, which is a multidisciplinary approach that integrates traditional nutrition with other fields, including biology, immunology, molecular biology, genetics, computer sciences, chemistry, biochemistry, mathematics, engineering, and technology sciences. For measuring the results of the body’s biochemical activities and figuring out Living systems’ dynamic, multi-parametric metabolic response to pathological stimuli, metabolomics can be a very useful instrument. Numerous metabolomics techniques exist, including emerging capillary electrophoresis (CE), gas chromatography mass spectrometry (GC–MS), nuclear magnetic resonance spectroscopy (NMR), liquid chromatography mass spectrometry (LC–MS), inductively coupled plasma mass spectrometry (ICP-MS), and some targeted HPLC-based assays. Metabolomics can help in understanding the metabolism of diets (protein, carbohydrate, and fat) and the pathways of precise nutrition. Metabolomics can revolutionize poultry nutrition strategies, optimizing health, growth performance, and metabolic efficiency by decoding biochemical interactions between diet, metabolism, and physiology. This review aims to highlight methodologies for integrating metabolomic data into precision feeding systems, ultimately enhancing sustainability, reducing production costs, and improving poultry welfare.

## 1 Introduction

The significant environmental factor influencing the growth, development, health, and profitability of chicken production is nutrition. Poultry feeds can account for as much as 70–75% of total production expenses (1, 2). Because of the unique gastrointestinal system structure, the poultry diet is very different from that of other livestock. For chicken nutrition, energy, fat, minerals, fiber, vitamins, amino acids, and water are essential, and feeds must have these nutrients in amounts that vary according to the species and age of the bird (3). Broiler precision nutrition necessitates the use of instruments that can detect individual or collective amino acid imbalances and the understanding of how to create more digestible proteins for creative feeding regimens that are tailored to the changing requirements of the animals (4).

Metabolomics is a discipline within omics that emerged in the mid-1990s, originating from the concept of metabolome. Metabolomics involves the qualitative and quantitative analysis of tiny molecular metabolites (≤1,000 Da) in cells, tissues, organs, or organisms, which indicate the metabolic pathways of endogenous metabolites influenced by internal and external stimuli (5). Metabolomics, characterized by high throughput and sensitivity, has emerged following genomics and proteomics, representing the pinnacle of the genomic trio (6). Metabolomics can be classified into two categories: non-targeted metabolomics and targeted metabolomics, based on the analytical scope. Non-targeted metabolomics focuses on all endogenous metabolites within an organism, whereas focused metabolomics concentrates on certain target molecules (7).

**Figure 1:**
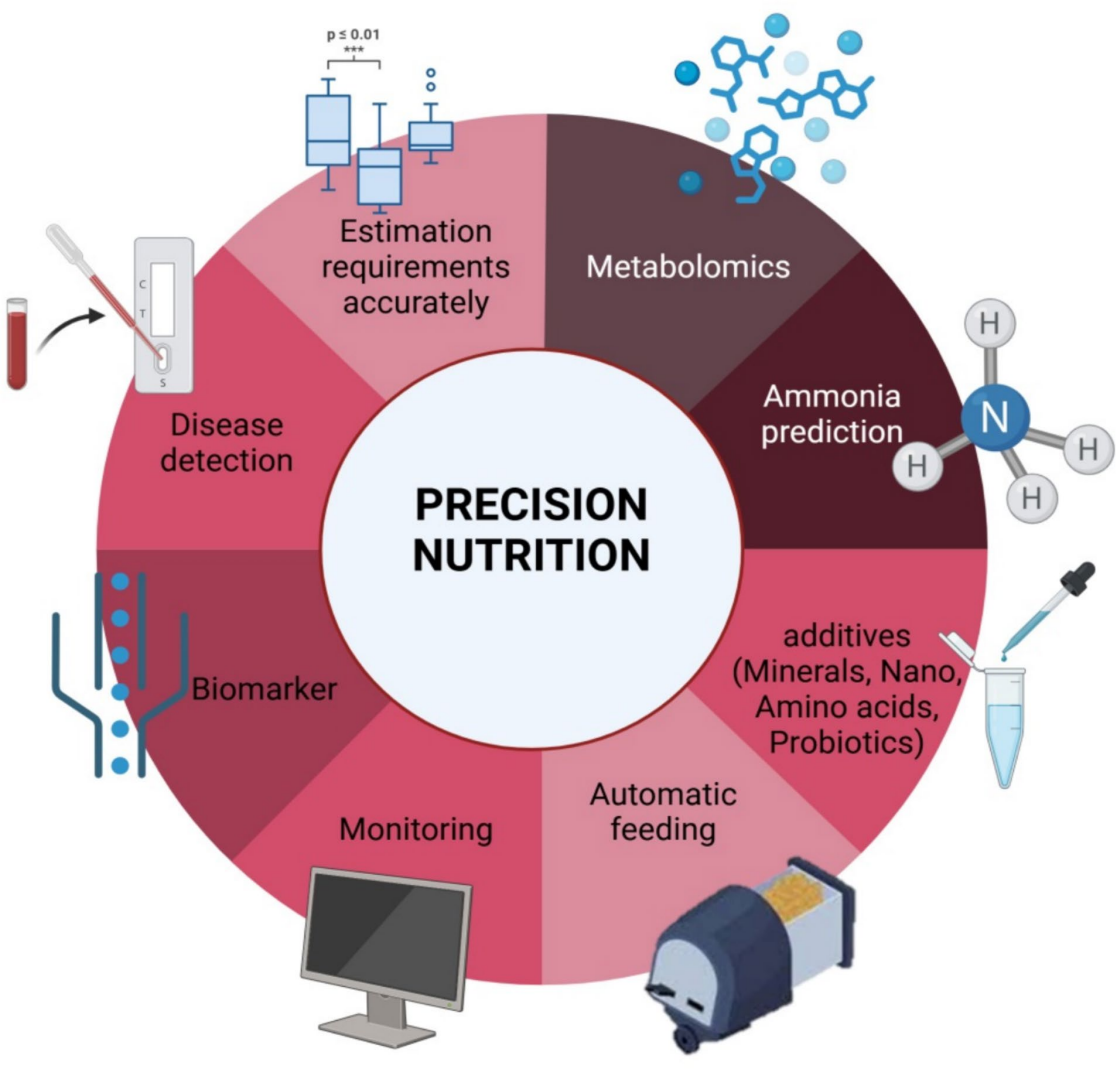
Main elements of precision nutrition.

Precision nutrition is an integrated strategy that amalgamates traditional nutrition with other domains, such as life sciences, immunity, molecular genetics, computational chemistry, biochemistry, mathematics, engineering, and practical sciences (8). Also, Modern feeding techniques and strategies, such as precision feeding and nutrition, can reduce nitrogen excretion by 30% compared to group phase feeding (9). Thus, it seeks to precisely match the nutritional requirements of animals with modified diets and necessitates both specified animal nutrient requirements and a comprehensive and precise nutritional database for every item (10). This idea, which is fundamentally related to animal husbandry methods, is essential to maximizing feed efficiency for the highest possible financial gain and minimizing losses. Ironically, though, despite its long history, precise nutrition has not yet been fully applied in the production of broilers. The nutritional needs of rapidly growing broilers change over time, and blending diets or multiphase feeding alone are insufficient to meet daily fluctuations (11). The operation of modifying and feeding diets to guarantee that an animal’s dietary nutrient supply matches its nutrient requirements at any given time is known as precision nutrition (12). Summary of main elements of precision nutrition in Figure 1.

Metabolomics measures a lot of small-molecule compounds in cells, organs, and biofluids using sophisticated analytical chemical techniques. A significantly more comprehensive picture of system-wide biology and metabolism is being painted by scientists thanks to the capacity to quickly identify and measure hundreds or perhaps thousands of metabolites in one sample. Researchers may now concentrate on quantifying the results of intricate, challenging-to-understand genetic or epigenetic interactions with environmental interactions thanks to metabolomics. Additionally, its usage in livestock monitoring and research is growing (13, 14). Metabolomics is used in studying metabolism in microbes, plants, and animals, revealing gene function, molecular breeding, and discovering phytochemicals for pharmaceuticals and animal development pathways, as well as disease and drug discovery investigations (15). Due to the growing interest in gut flora, commonly known as the “microbiome,” and the possibility of industrial uses such as biofuel production (16), microbial metabolomics is also currently of significant interest (17).

A newly established domain of “omics” research known as “metabolomics” meticulously analyzes small molecule constituents in biological matrices such as tissues, cells, and biofluids with advanced analytical chemistry methodologies (18). Metabolomics can be a very useful tool for measuring the results of the body’s biochemical activities and assessing the dynamic, multiparametric metabolic response of living systems to pathological stimuli. Since chicken is among the most eaten meats globally, the bulk of metabolomic surveys conducted on all avian types were on this species (19). Nonetheless, a modest number of metabolomics investigations have been conducted on nestling birds, domesticated animals, and meat (20, 21). Physiology, nutrition, health, production, reproduction, human health, and poultry products are some of the categories into which poultry metabolomics research can be divided (13).

**Figure 2:**
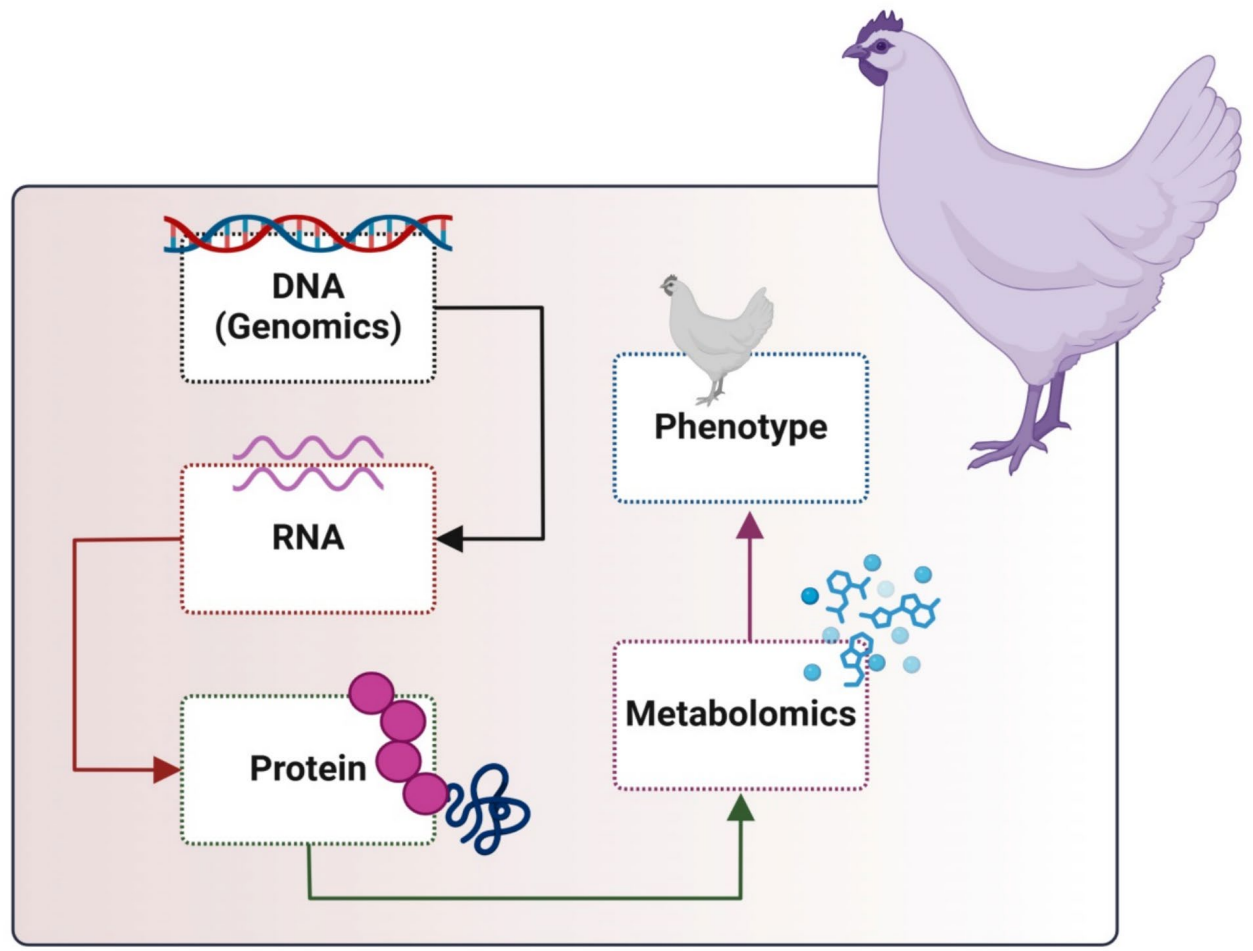
Metabolomics in the cycle of omics in animal cells.

Metabolomics is extensively utilized across multiple domains, including illness diagnosis and mechanisms (22, 23), medication development (24, 25), nutritional science (26, 27), microbial research, and nutraceutical science (28). Metabolomics has been increasingly utilized in physiology, pathology, nutrition, and feed safety within poultry research. Metabolomic study of poultry blood, muscle, kidney, liver, and egg products can indicate health state, meat quality, egg quality, and pathogenic causes (29). In broiler breeding, increasing feed efficiency (FE) is among the primary goals. Breeders have been looking for biomarkers for the different selection and enhancement of feed efficiency features because it is challenging to measure these qualities directly. The “bridge” connecting the genome and phenome is the metabolome (Figure 2). More phenotypic variance may be explained by the metabolites, which can also be used as appropriate biomarkers to choose feed efficiency features (30). As demonstrated by certain attempts to incorporate new algorithms and construct templates for phenotype prediction, metabolomics techniques have been improving, particularly in the operation of statistical data analysis. However, using meat metabolome data and metabolomics that target the metabolomes of animal blood and meat exudate would support meat quality prediction as a simple technique (31). The main objectives of the present review are to focus on metabolomics and its impact on growth and efficiency to improve poultry production.

## 2 Overview of metabolomics (approaches and techniques)

Metabolomics, an increasingly prominent method, is distinguished from other “omics” fields by its molecular weight of less than 1,500 daltons, which is lower than that of most proteins, DNA, and other biological macromolecules (32). The term “metabolome” refers to the collection of small molecules found in a certain biological sample. Researchers employ complementary methodologies such as gas chromatography mass spectrometry (GC–MS), nuclear magnetic resonance spectroscopy (NMR), liquid chromatography mass spectrometry (LC–MS), emerging capillary electrophoresis (CE), inductively coupled plasma mass spectrometry (ICP-MS), and various targeted HPLC-based assays, as a single tool or separation technique cannot detect every metabolite within a metabolome (18, 33). The progression of modern metabolomics techniques may enable a broader range of study disciplines to distinctly separate, identify, and quantify molecules with precision (19).

The last and most similar stage to the phenotype in the “omics” cascade is metabolomics (34). Metabolomics, which is referred to as “the apogee of omics” since it is the closest to the phenotype, has the benefit of acting as a fast indicator of biochemical activity (35). Although the number of studies using the term “metabolomics” has skyrocketed over the past 20 years, tools for thorough metabolomics analysis are still in their infancy when compared to different “omics” like “proteomics.” Numerous metabolites are included in the metabolome.

Metabolomics represents a groundbreaking approach in poultry nutrition, offering a unique opportunity to directly link metabolic processes to phenotypic outcomes. While still in its developmental stages compared to other “omics” fields, the ability to identify and quantify metabolites with precision holds great promise for optimizing growth, health, and feed efficiency. By integrating advanced metabolomic techniques, we can unlock new insights into gut microbiota, immune function, and stress responses, ultimately paving the way for more sustainable and individualized feeding strategies in poultry production.

### 2.1 Nuclear magnetic resonance spectroscopies (NMR)

The NMR may give a “holistic view” of the metabolites in a complicated sample; its application in metabolomics is particularly beneficial. Among its numerous benefits is its non-destructive nature, which allows a sample to be reused or remeasured multiple times. It offers comprehensive structural details that may aid in the identification of unidentified metabolites. It involves little sample making, like separation or derivatization, and is independent of analyte polarity (36, 37). Because it offers a repeatable correlation between signal intensity and metabolite concentration, NMR is useful for metabolite quantification. Although NMR can employ a variety of nuclei, proton is the most utilized nucleus because of its large abundance and consequently superior sensitivity. Additional nuclei, such as 13C and 31P, can be utilized for flow analysis and phosphorus-containing compounds, respectively (38). NMR metabolomics has weak sensitivity, making it unsuitable for low-abundance metabolites (37), and signal overlap makes it difficult to identify distinct particles in complex mixtures (39). Prior sample separation can enhance NMR identification and reduce sample complexity, but as discussed in the next sections, it will also decrease method sensitivity (Table 1).

**Table 1:** Comparison of GC–MS with other poultry analysis techniques, focusing on sensitivity, cost, and other key factors.

Technique	Sensitivity	Cost	Complexity of sample preparation	Analysis time	Utilizations in poultry	Limitations	References
GC–MS	High (ppb-ppt)	High	Moderate-High (Derivatization is often needed)	30–60 min	Pesticides, volatile contaminants, fatty acids	Limited to volatile/thermally stable compounds	(43)
LC–MS	Very High (ppt)	Very High	Moderate	20–40 min	Antibiotics, hormones, and polar residues	Matrix effects, high instrumentation cost	(56)
HPLC	Moderate (ppm-ppb)	Moderate	Moderate	20–30 min	Vitamins, mycotoxins, and some drug residues	Lower sensitivity vs. MS methods	(44)
ELISA	Low-Moderate (ppm)	Low	Low	<30 min	Rapid screening for pathogens, drug residues	Qualitative/semi-quantitative, cross-reactivity	(29)

### 2.2 Mass spectrometry (MS)

**Figure 3:**
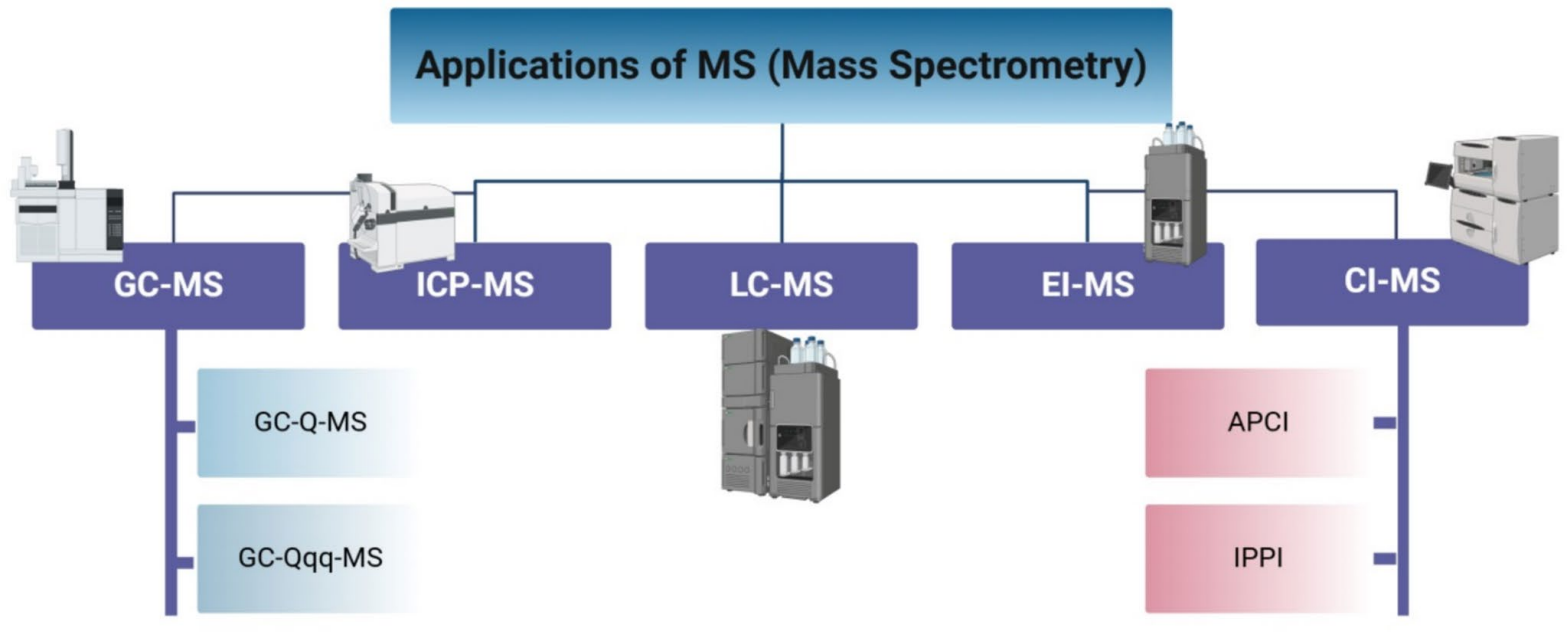
Applications for mass spectrometry.

According to Emwas et al. (40), MS is generally more sensitive than the NMR technique, with a lower limit of detection for most metabolites being in the femtomole range as opposed to the low nanomole range for NMR. In contrast to NMR and MS, it is a destructive technology, meaning that the selected sample is often lost after the end of analysis and cannot be utilized again. According to Letertre et al. (41), NMR usually identifies high tens to slightly over 100 metabolites, but MS may measure hundreds of metabolites with different characteristics and concentrations at the same time. MS can be used in conjunction with a separation procedure or as a stand-alone platform by putting the sample straight into the mass spectrometer. Direct sample analyses using a mass spectrometer have numerous drawbacks, even though they offer the quickest analysis time and could cover a wide variety of metabolites (42). One of these drawbacks is that molecules compete with one another for ionization, which causes less effectively ionized molecules to be suppressed by more highly ionized ones. The absence of a separation dimension, which frequently offers an extra layer of assurance regarding the identity of a metabolite, is another drawback. Thus, a mass spectrometer, data analysis tools, and a separation technique make up a conventional MS-based metabolomics platform. Metabolome analysis is further impacted by the differences in ionization and detection capabilities among mass spectrometers. There are many types of applications of MS (Figure 3).

### 2.3 Gas chromatography mass spectrometry (GC–MS)

In a recent study, Munjal et al. (43) found that GC–MS is a flexible analytical method due to its exceptional capacity to separate substances, robustness, responsiveness, high level of selectivity, and reproducibility results. With established analytical methods for performing the metabolite analysis of substances present in medicinal or herbal plants, including amino acids, sterols, hormones, sugars, catecholamines, aromatics, fatty acids, etc., this methodology is one of the most systematic in the field of metabolomics. Because it offers higher chromatographic accuracy than liquid methods, it provides a few advantages over LC–MS (44).

GC–MS is an invaluable tool in metabolomics due to its extensive database and Electron Ionization technique, which allows for the study of a wide range of compounds with just one column, making it suitable for untargeted research (45). Metabolomic studies can be used in several pathways to characterize volatile constituents, including simple headspace injections (46), thermal desorption of volatile chemicals on adsorption matrices in the GC–MS system, usually using active adsorption methods (47) and Solid-Phase Microextractions (48), or passive adsorption methods that use materials with a capacity that is relatively larger than SPME fibers (32). Furthermore, the use of GC–MS to carry out metabolomic analyses of primary metabolites is restricted to plant research and is less common. The most widely utilized MS detector in metabolomic studies, including GC, is Electron Ionization Mass Spectrometry (EI-MS), also one of the many practical ionization sources used in the field of metabolomics. Mass spectra should be repeatable across instruments with different mass analyzers, and all electron ionization instruments are ionized at 70 eV (49). Also, in the context of metabolomics, the Electron Ionization approach is used more frequently than the Chemical Ionization technique. This could be explained by the fact that the chemical ionization technique falls within the soft ionization approach, which produces pieces that are not very sensitive and are therefore unusable for identification. However, the Chemical Ionization technique is only beneficial in targeted techniques because it is unable to produce many molecular ions (44, 50). Atmospheric pressure chemical ionization and atmospheric pressure photoionization (APCI and APPI, respectively) are examples of other soft ionization methods. These days, APCI resources are coupled with very high-resolution mass systems such as Time-of-Flight (ToF) systems, Fourier Transform (FT) Orbitrap, and Ion Cyclotron Resonance (ICR) (43). GC is connected to various mass analyzers with varying accuracies and resolutions. Tiny mass resolution quadripartite mass filters, such as GC single quadruple (GC-Q-MS) and triple quadruple instruments (GC-QqQ-MS), are the most often used mass analyzers among the many GC–MS instruments. The primary benefits of quadruple-based systems typically include their greater dynamic range and significant responsiveness; nevertheless, one disadvantage of these systems may be their slower scan rates and reduced mass accuracy when compared to high mass resolution-based devices (43, 51).

### 2.4 Liquid chromatography mass spectrometry (LC–MS)

The combination of liquid chromatography (LC) and mass spectrometry (MS) is known as liquid chromatography and mass spectrometry (LC–MS) (52). Many metabolomic studies often involve a separation process before the mass spectrometry analysis (43). High Performance Liquid Chromatography (HPLC) is a versatile and effective separation method that enables the breakup of chemical entities with varying polar ranges utilizing either the gradient elution method (where the water-solvent ratio is kept variable all the process) or the isocratic elution method (where the ratio is kept constant all the procedure) (53). Water, acetonitrile, formic acid, and methanol are a few of the most often utilized organic solvents in LC. In general, isocratic elections are favored for simple samples. Compared to the previous elution procedure, gradient elution has the advantages of fast analysis, narrow peaks, and somewhat similar resolution (54). The three main components of an MS system are typically a mass analyzer, an ion source, and a detector. The source of ions’ primary function is to convert sample molecules into ions, and the mass analyzer resolves the ions before the detector measures them. Atmospheric pressure photoionization, chemical ionization, and MALDI are among the various options available as ion sources (43).

To enhance the coverage of metabolome even more, it is occasionally necessary to analyze both the positive and negative modes of ionization due to the diversity in the metabolites’ chemical characteristics. The recommended technique for metabolomic analyses based on LC–MS is without a doubt ESI. The rationale is that it is a gentle ionization technique, which helps with identification by producing a lot of ions by charge exchange in solution and often forming entire molecule ions. Reverse Phase Liquid Chromatography (RPLC) is an effective method for separating semi-polar molecules such as flavonoids, phenolic acids, alkaloids, and other glycosylated entities in LC-ESI-MS. In Hydrophilic Interaction Liquid Chromatography (HILIC), polar substances such as sugars and amino acids are eluted using columns such as aminopropyl columns (55). Even though high-polarity chemicals can be separated using Normal Phase Liquid Chromatography (NPLC), APCI-MS is found to work better with nonpolar organic mobile phases than ESI-MS. NPLC-APCI-MS is typically used to examine the hydrophobic lipids’ analytical profile, including triacylglycerols, sterols, and fatty acid esters. When doing metabolomic analyses on complicated materials, LC-ESI-MS has emerged as the method of choice (56). The benefits of integrating an HPLC system with MS include enhanced signal repeatability and MS responsiveness because of sample complexity reduction, which lowers matrix interference throughout the ionization process. Furthermore, improved separation by chromatography will yield trustworthy MS data because background noise is decreased. Ultra-Performance LC (UPLC) and capillary monolithic chromatography are two recent developments in LC that have significantly increased peak resolution and analytical time (57, 58).

Precision livestock farming (PLF) is one of the innovative technologies being increasingly adopted by the poultry industry to maximize and enhance bird productivity and welfare. PLF monitors animal productivity, environmental impacts, and health and welfare to assist farmers with livestock management. To provide an automated management system, PLF relies on the automatic collection, retrieval, and processing of data from many sources (59, 60). To achieve this, machine learning algorithms examine the data and produce models for risk assessment or forecasts (61). This enhances animal welfare and productivity by enabling the farmer to identify and address health concerns early on (62, 63). Metabolomic data could provide detailed insights into the physiological status of animals. For example, changes in metabolite profiles might indicate stress, disease, or nutritional deficiencies before visible symptoms appear. That could allow for earlier interventions, which is a key goal in PLF (60).

## 3 Nutritional requirements and metabolomic profiling in poultry

Populations of comparable animals’ nutritional needs are often established based on their age, physiological condition, genetics, and sometimes sex. Individual differences within animals cannot be handled by the population-feeding technique (12), and as a result, specific needs based on genetics or nutritional status, environmental stress, and animal health-related circumstances may be disregarded. Precision livestock production relies heavily on individual animal management (64), which can help avoid overfeeding, especially in developing pigs (12). However, it is debatable if the poultry industry can effectively manage individual birds with accuracy (8). Overfeeding protein leads to economic losses, increased nitrogen (N) environmental load, and ammonia emissions (65).

According to Cambra-López et al. (4), poultry require modified amino acid levels that are optimally mixed to meet each amino acid’s requirements without excess or shortfall. Compared to swine or cattle, broilers are the most effective animals in turning proteins into meat (66, 67); nonetheless, their nitrogen retention is tiny, ranging from 57 to 60% (68). As a result, over half of the protein that poultry consumes is eliminated. Furthermore, gut health may be adversely affected by Protein that has not been digested and its metabolites produced during the fermentation of protein (69). In addition to being utilized by unwanted pathogenic bacteria, Protein that is not digested in the distal gastrointestinal system can compromise gut integrity and function (70). Additionally, amino acids must be catabolized in the liver if they are accessible to excess or are not balanced adequately. This finding is in the production and release of ammonia gas, which is extremely poisonous (4). Adenosine triphosphate (ATP) is necessary for the step of deamination of non-essential amino acids in the tissue of the liver and the secretion of ammonia as uric acid in chickens, both of which are expensive for the animal and require three ATP molecules for each nitrogen molecule expelled (71). All of this exacerbates the adverse effects of poultry meat production on the environment and significantly impairs the productivity and health of the animals. Balanced feed with maximum amino acid digestibility that are adapted to the needs of each animal over time need further study, even though nutritionists today use methods for evaluating animal amino acid requirements and optimized feed supplies combined with modeling techniques that can aid in the process (based on real ileal digestible amino acids, rather than conventional crude protein estimation or fecal total amino acids) (72, 73). Some amino acids are glucogenic, others ketogenic. So, their balance might influence energy metabolism. If there’s an imbalance, the bird’s body might need to compensate by breaking down more proteins or altering energy production, leading to changes in metabolites like glucose, ketones, or urea (4, 68). Additionally, diet affects gut bacteria, which in turn produce metabolites like short-chain fatty acids. If amino acid balance is off, maybe the microbiome changes, leading to different metabolomic profiles. That could be a biomarker for gut health or nutrient absorption efficiency (70, 71). To achieve the best possible fit of amino acid supply to animals’ dynamic requirements, precision nutrition in broilers necessitates the use of tools that can recognize deficiencies or problems individually or in groups, as well as an understanding of how to obtain more palatable and usable proteins or even design imaginative feeding programs (4).

This section underscores the limitations of population-based feeding in poultry, which fails to address individual nutritional needs. I believe precision livestock production, using tools like metabolomics, can optimize feed efficiency and reduce environmental impact by tailoring nutrition to each bird’s specific requirements. Imbalances in protein and amino acids harm both health and productivity, so further research into precision nutrition and metabolomics is crucial. Understanding how diet affects gut microbiota could also offer biomarkers to improve gut health and nutrient absorption, ultimately enhancing poultry welfare and performance.

## 4 Impact of dietary interventions on poultry metabolome

Numerous factors, including protein production rate, loss of protein, consumption, and rate of metabolism, influence serum total protein. Zinc is involved in several biochemical processes, including protein synthesis, glucose metabolism, and the enzyme system (74). Additionally, earlier studies showed that dietary zinc supplements had a substantial impact on laying hen performance, serum total protein, albumin, glucose, and immunoglobulin (75, 76). The body breaks down proteins to produce urea nitrogen. The improvement in protein catabolism is indicated by the rise in nitrogen content. According to Qi et al. (74), serum urea nitrogen showed a noteworthy upward trend when supplemented with 80 mg/kg MHA-Zn. Furthermore, some research has shown that the organic zinc group reduced the levels of low-density lipoprotein (LDL) cholesterol and serum triglycerides when compared to the group that did not take any supplements (77). Also, the 1H-NMR-based approach, according to Qi et al. (74), demonstrated metabolic alterations and identified important metabolic pathways that represent various zinc sources; the 2nd of these pathways, glutathione metabolism and glycine, threonine, and serine metabolism, demonstrated a strong correlation with Zn indications. These two routes and metabolite indicators are associated with energy, amino acid metabolism, and oxidative stress. The liver of the 40 mg/kg MHA-Zn collection had higher levels of threonine than the base group in the glycine, serine, and threonine pathways (74).

**Figure 4:**
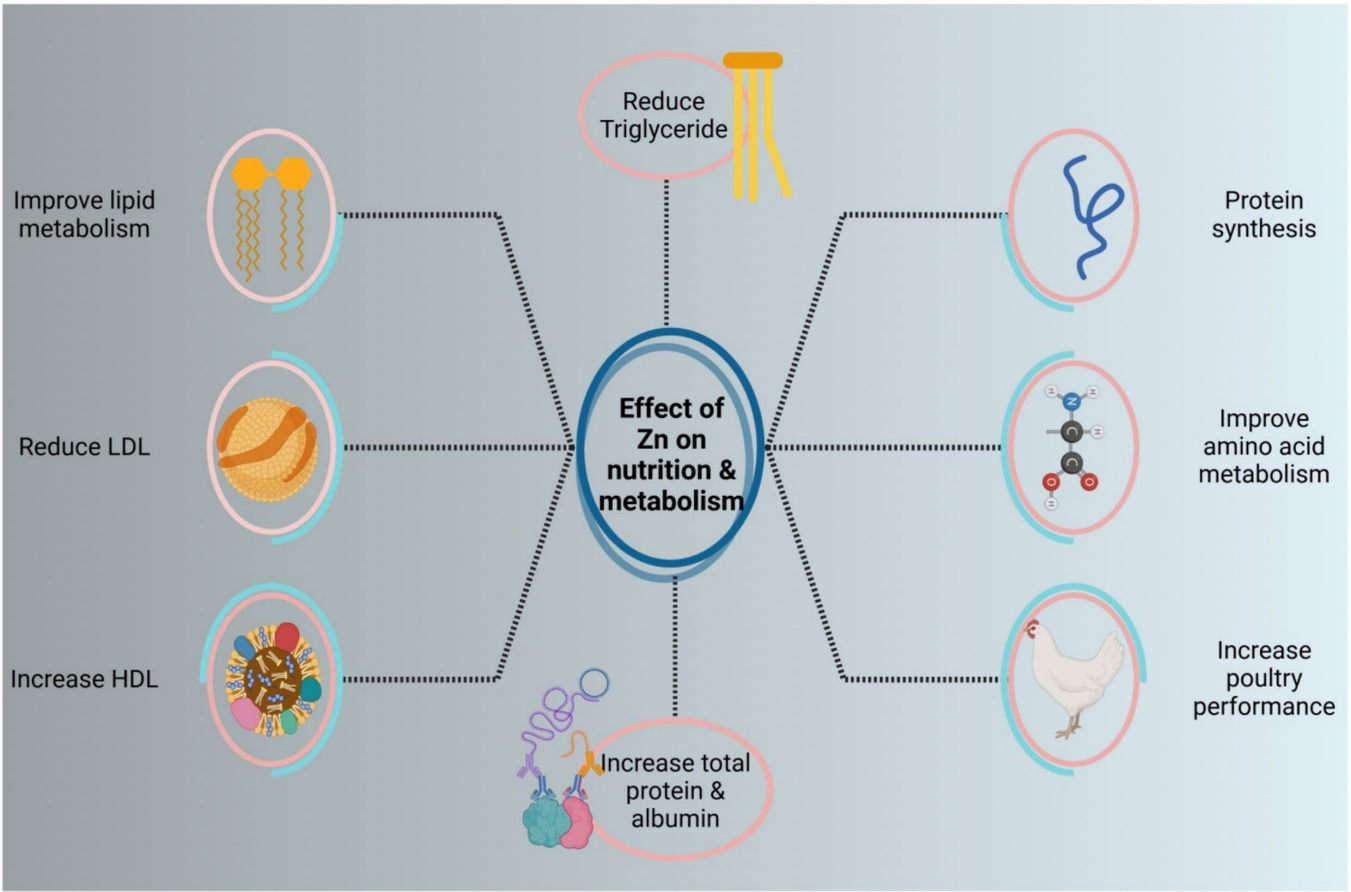
The role of Zn in metabolism and nutrition.

Also, Wessels et al. (78) demonstrated that zinc can lower triglycerides and influence the metabolism of fat and glucose formation. In another study, adding zinc to the diet boosted high-density lipoprotein cholesterol (HDL-C) and lowered triglyceride (TG) content, confirming zinc’s beneficial influence on lipid metabolism (79, 80). In many layers of broilers, supplementation of organic zinc can raise the number of enzymes and gene expression related to lipid metabolism. This is because the liver’s fat synthesis is more vigorous in late stages of egg production, which increases the risk of inflammation (81, 82). According to Qi et al. (74), MHA-Zn continues to influence lipid metabolism because of the elevated betaine and decreased choline levels. The role of Zn in metabolism and nutrition is shown in Figure 4.

**Figure 5:**
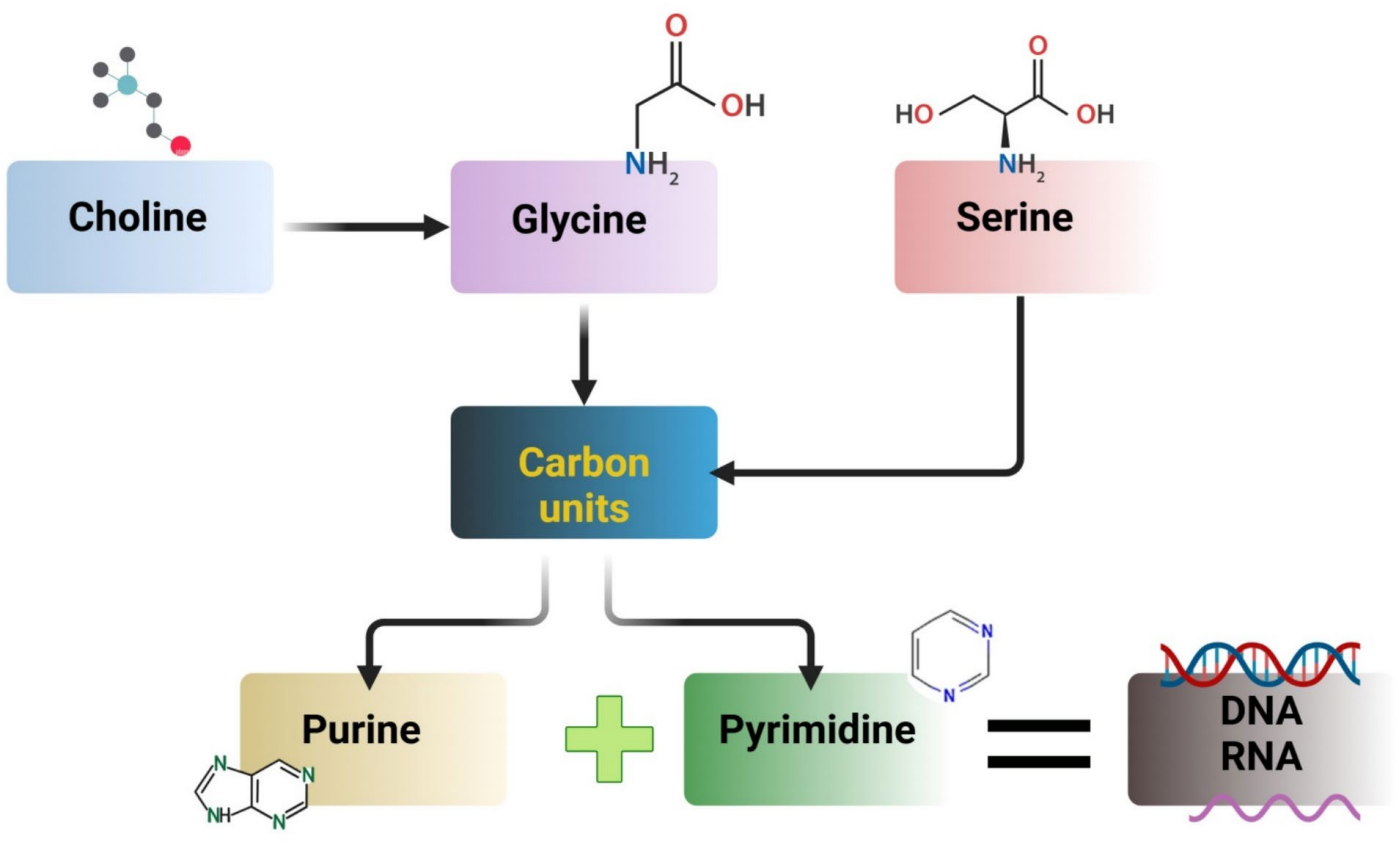
Amino acids metabolism in poultry.

As an essential amino acid for chickens, glycine contributes to the production of uric acid, where three metabolic processes involving serine, threonine, and choline contribute to glycine formation (74). When viewed on an equimolar basis, serine has been discovered to be equally effective in fulfilling the works of glycine (83). Additionally, the metabolomics of serine and glycine yields units of carbon that are used as building blocks for pyrimidine and purine biosynthesis. A necessary amino acid and a significant methyl donor, methionine (Met) plays vital functions in transsulfuration and 1-carbon metabolism. The addition of methionine (Met) may increase flow during the transsulfuration process, which in turn increases the formation of S-adenosyl-Met (SAM), which uses ATP to make taurine and the antioxidant glutathione (GSH). According to Maddocks et al. (84), increased antioxidant synthesis may aid in lowering oxidative stress and inflammation brought on by the liver’s generation of reactive oxygen species. Furthermore, by *de novo* ATP generation, serine aids in the Met cycle (85). By producing the Met through its metabolite betaine, choline also aids in the Met cycle (86). N, n-dimethyl glycine is the product of this reaction and undergoes two stages of metabolism: first, it is converted to creatine and subsequently to glycine (87). In Figure 5, some amino acid metabolism in poultry is shown.

This section highlights the significant impact of dietary interventions, such as zinc supplementation, on poultry metabolism. I believe this demonstrates the critical role of specific nutrients in regulating metabolic pathways, improving overall health, and optimizing performance in poultry. Zinc, in particular, affects protein synthesis, lipid metabolism, and oxidative stress, all of which are crucial for poultry production. Understanding these metabolic alterations through advanced techniques like NMR is key to refining dietary strategies for poultry health and productivity.

## 5 Gut health and metabolomic responses

While “microbiota” and “microbiome” are frequently used interchangeably, distinct differences exist between the two terminologies. Microbiota refers to the live microorganisms present in a specific habitat, such as the oral and gastrointestinal microbiota. The microbiome denotes the aggregate of genomes from all microorganisms within an ecosystem, encompassing not only the microbial population but also the structural components, metabolites, and environmental circumstances (88). The microbiome includes a broader spectrum than the microbiota. The comprehension of the host-microbiota interaction has facilitated the creation of microbiota-based therapeutics, such as fecal microbiota transplantation and bacterial regulation, enhancing clinical outcomes in conditions like *Clostridium difficile* infection, diabetes, and inflammatory bowel disease (89). Furthermore, the microbiome drives a metabolome that influences the avian host’s body weight and energy balance, as well as host intestine metabolism and immunity (90). The microbiome is then shaped by the mucosal immune system, and the nutritional state of the host affects the composition of the commensal microbial population and aspects of the host defenses (91). The microbial community in the chicken gut is a multilayered, dynamic system with a homeostasis state and a certain capacity for structural resilience (92, 93), even though the species composition and metabolic functions of the gut microbial community can be readily changed by diet, antibiotic ingestion, pathogen infection, and other host-and environment-independent events (94, 95).

According to Maritan et al. (96), the complexity of an animal’s nutritional relationships is greatly increased by the gut microbiota, which is fed by the host and provides essential nutrients as well as participates in the host’s physiological systems, including immunological defense. There is growing evidence that describes how the intestinal microbial community’s composition and role affect the nutritional value of feed, and that feed influences the microbiota and its extensive grouping of bacteria’s genes in the intestinal microbiota (90, 93). With the microbiome being directly linked to the bioconversion of feed components to alterations in host physiology, metabolism, and immunity, diet plays a vital role in forming and controlling the gut microbiota. Depending on the quantity and variety of the resulting microbial composition and the generation of resulting metabolites, dietary changes can cause a substantial change in microbial composition in as little as 24 h (97). This change may or may not be advantageous to the host physiology (98).

The host’s exposure to bioactive dietary ingredients and their possible health effects can be altered by the gut microbiota’s metabolic activity. Furthermore, certain components of functional feed affect the gut microbiota’s growth and/or metabolic activity, which in turn affects the microbiota’s composition and process (99). Therefore, the gut microbiota might influence the biological activity of other dietary molecules and be a target for nutritional interventions aimed at enhancing health (100). Nearly every vitamin in the feed is necessary for maintaining a healthy immune response; thus, both inadequate and excessive intakes can impair immunological function. It has long been recognized that the immune system of poultry is influenced by its diet, dietary nutrients, and dietary variables (101). More than two decades later, it is still unclear how the gut microbiota and host nutritional mechanisms interact to facilitate these avian immunohistology processes (99). The intestinal immune system can also ascertain the metabolic condition of the microbiota by detecting microbial metabolites via their pattern recognition receptors (PRRs) (102, 103). The microbiota breaks down metabolites from the host and diet through a variety of metabolic pathways, which then impact various components of the intestinal immune system. The microbiota uses indigestible fibers to create short-chain fatty acids (SCFAs; acetate, propionate, and butyrate), which have several anti-inflammatory effects on chicken immune cells *in vitro* and *in vivo* (104, 105).

Understanding the role of microbiota in poultry health requires not only studying microbial composition but also identifying and quantifying the metabolites they produce. Small-molecule metabolites, such as short-chain fatty acids (SCFAs), indole compounds, and secondary bile acids, are among the most functionally significant products of microbial activity. These compounds influence a wide range of physiological processes in the host, including gut barrier function, immune regulation, and nutrient absorption (90). To accurately assess the presence and concentration of these metabolites, advanced analytical techniques are employed. Gas chromatography–mass spectrometry (GC–MS) and liquid chromatography-mass spectrometry (LC–MS) are widely used due to their sensitivity and precision. These tools allow researchers to detect even trace amounts of metabolites in intestinal contents, blood, or feces, enabling a clearer understanding of how diet, microbiota, and host health are interconnected (95–97).

For example, increased levels of butyrate—identified through LC–MS analysis—have been correlated with improved gut integrity and reduced inflammation in broilers. Similarly, elevated concentrations of indole derivatives have been linked to stronger immune tolerance and lower susceptibility to intestinal infections (102). By combining metabolic profiling with microbiota analysis, researchers can gain deeper insights into the functional contributions of the gut microbiome. This opens the door to targeted nutritional interventions that enhance the production of beneficial metabolites, ultimately improving poultry growth performance, feed efficiency, and disease resistance.

This section underscores the critical relationship between gut health and the metabolomic responses in poultry. I believe the complexity of the gut microbiome and its profound influence on the host’s metabolism, immunity, and overall health cannot be overstated. The gut microbiota is not just a passive participant but actively interacts with dietary components to shape the poultry’s physiological state. Understanding how dietary interventions impact microbiota composition and its metabolic activity opens avenues for targeted nutritional strategies that can improve both poultry health and production outcomes. The emerging field of microbiota-based therapeutics, particularly in poultry, promises to enhance gut health and immune response, leading to more efficient and sustainable poultry farming practices.

## 6 Antioxidant status and immune function

During cellular metabolism, most oxidants are produced by the mitochondria of living cells. In addition to cellular metabolic processes, some exogenous sources, including feed containing oxidized lipids and fats, can also produce ROS (106). Oxidant damage and decreased growth performance in poultry under heat stress conditions have been linked in many studies (107, 108). Due to intestinal injury and metabolic disruptions that impair feed utilization, OS can have a significant effect on the feed conversion ratio (109). Furthermore, Reprogramming the metabolism, Erythroid 2-Related Factor two (Nrf2) is a nuclear factor that directly alters intermediate metabolism and organizes genes that generate protective proteins that direct the breakdown and reform of impacted macromolecules under stressful circumstances (110). ROS are already produced by the body’s regular metabolism and biochemical processes. However, the birds’ antioxidant defenses are overpowered when they are exposed to these poisons, which dramatically raise ROS levels (111). ROS are metabolic byproducts of some microorganisms. Superoxide radicals and hydrogen peroxide are two types of ROS that bacteria can create during aerobic respiration (112). Heat stress can increase ROS levels, lowering the GSH/GSSG ratio, requiring more glutathione production. Enzymes involved in glutathione synthesis may be upregulated (111). Therefore, the metabolomics approach would look at the levels of glutathione and related metabolites. They might use techniques like mass spectrometry or NMR to measure these. Also, the pathways involved in glutathione metabolism—like the synthesis pathway (involving glutamate, cysteine, and glycine), the enzymes glutathione synthetase and gamma-glutamylcysteine synthetase, and the recycling enzyme glutathione reductase (113).

The primary energy source for colonic epithelia, butyrate or butyric acid, is essential for preserving colonocyte homeostasis and the formation of gut villus morphology (95), as well as for boosting intestinal barrier integrity, improving growth performance and carcass quality attributes, and reducing Salmonella immigration in poultry (114). Moreover, most of the published research describes how nutrition affects physiological functioning, particularly the immune system, and how intestinal microbiota interacts with feed. Most research has found that certain nutrients (protein, fatty acids, and carbohydrates) directly affect the immunological system without figuring out how they affect the gut microbiota directly, which results in A side effect on antibodies (115). The microbiota in the gut is crucial for regulating the host’s immune system (116), metabolism (117), and brain function (118). But also, other physiological functions and traits, including the gut-bone axis (119), the gut-liver axis (120), and the gut-muscle axis (117), that are believed to be entirely dependent on the bird’s genetic program. It makes sense that the macrobiotic is engaged in a wide range of metabolic interactions with its avian host, including several metabolic pathways that control several host microbiota’s metabolic, immunological, and communication axes, like the synthesis of SCFA, which physiologically links to several systemic organ systems (121). However, a more comprehensive knowledge of the gut microbiota’s impact on chicken nutrition would require systems biology studies of the metabolic and immunological links of the poultry’s gut to the gut microbiota (122).

The interplay between antioxidant status, immune function, and oxidative stress in poultry is central to maintaining health and optimizing performance, particularly under environmental stresses like heat. I believe this section highlights the critical role of dietary antioxidants and gut health in mitigating oxidative stress and improving the poultry immune system. The relationship between ROS production, glutathione levels, and intestinal health suggests that better understanding and management of these pathways can lead to improved feed conversion rates and overall poultry welfare. Moreover, as the microbiota significantly influences immune function and metabolism, I see an opportunity for innovative nutritional strategies that balance antioxidant intake, gut health, and immune function, thus enhancing poultry production efficiency and sustainability.

## 7 Metabolomics for growth and feed efficiency

According to Mahoro et al. (123), growth signs in poultry are also associated with significant economic features, and conventional breeding techniques have proven effective in enhancing growth qualities genetically (124). Previous studies on these characteristics have concentrated on identifying genetic markers linked to growth qualities (125, 126). These markers include MLNR, which codes for the gut hormone motilin receptor; TGF*β*3, which codes for conversion growth factor β (127); ACTA1, which codes for skeletal *α*-actin (128); and the mediator complex gene MED4, which is necessary for transcriptional activation (117). The intricate phenotype of poultry growth, which is also connected to sex, nutritional conditions, and illness state, cannot be entirely explained by host genome information (129–131). Since metabolites serve as crucial bridges between the genome and growth processes, the evolution of metabolomics technology has made available new pathways for studying host physiology (132).

Utilizing nuclear magnetic resonance and chromatography-mass–mass spectrometry, metabolomics is the technique of identifying and quantifying all small-molecule metabolites (<1,000 Da) in biological samples. According to Urgessa and Woldesemayat (133), these metabolites reflect the differences in animal genetics that are translated into the growth performance through the change in metabolic metabolites. Metabolomics’ findings about the synthesis and metabolism of small molecules can more precisely and directly represent the physiological condition and performance of the animals (134). Therefore, metabolomics can offer fresh perspectives on the examination of animal characteristics and could be used to find small-molecule metabolites that have a close relationship with indicators for feed intake (135), growth performance (136), meat quality (137), and disease (138). Serum uric acid and residual feed intake were found to be strongly positively associated in a prior study on the growth phenotype of chickens (139). Metabolomic biomarkers offer a transformative approach to poultry breeding, enabling precise selection for health, productivity, and sustainability. While technical and logistical challenges persist, integration with emerging technologies promises accelerated genetic gains and improved flock resilience, aligning with global demands for efficient and healthy poultry production (124). Poultry breeders use traditional breeding methods like phenotypic data and genetic markers, but metabolomics could provide a more direct view of physiological states, enabling the selection of birds with desirable traits (129). Additionally, Breeders can screen birds for biomarkers, such as metabolites indicating better immunity, and select birds with efficient nutrient utilization, thereby reducing costs and improving feed conversion (131). Additionally, the combination of metabolome and gut microbiome investigations showed that some cecal bacteria stimulated growth through conjugated linoleic acid (bovinic acid) (140). According to Zhang et al. (141), microbes and *Sphingomonas* are abundant in the cecum in hens and enhance growth performance by controlling fat metabolism. Body weight traits have a direct effect on the financial advantages of broilers and are a crucial indicator of chicken growth performance attributes (142). In redox reactions such as the TCA cycle, amino acid breakdown, mitochondrial respiration chain electron transfer, and fatty acid oxidation, all involve riboflavin, which participates in electron transfer as flavin mononucleotide and flavin adenine dinucleotide (143). According to Wang et al. (144), who assessed riboflavin adding in broiler diets under various nutritional circumstances, the ideal amount was 3.6 mg/kg supplied during the first four weeks of life. Chronic inflammation can significantly limit chicken growth, according to numerous earlier research studies (140, 145). The chicken jejunum’s markedly elevated production of TLR4, NF-κB, MyD88, and other cytokines compromised the intestinal barrier’s integrity, which in turn prevented the chicken from growing (146).

One of the primary objectives of broiler breeding is to increase feed efficiency, and Su et al. (30) proposed 10 metabolites that might be used as possible breeding indicators, including 7-ketocholesterol, epsilon-(gamma-glutamyl)-lysine, dimethyl sulfone, gamma-glutamyltyrosine, L-homoarginine, testosterone, 2-oxoadipic acid, adenosine 5′-monophosphate, adrenic acid, and calcitriol. “Feed-saving broilers.” According to a prior study, the fat line had a much greater residual feed intake (RFI) and feed conversion ratio (FCR) than the lean line (147). One possible biomarker for the inflammatory response was epsilon-(gamma-glutamyl)-lysine, which is a member of the type of chemical molecules known as glutamine and derivatives (148). Also, Yang et al. (149) found that animals with great feed efficiency exhibit fewer inflammatory reactions. Epsilon-(gamma-glutamyl)-lysine was found to be more abundant compared to the thin line, the fat line. This finding suggests that the fat line may have a larger inflammatory response and, as a result, worse feed efficiency. Consequently, it was proposed that plasma epsilon-(gammaglutamyl)-lysine might be used as a biomarker to choose feed efficiency features. Tyrosine (Tyr) is derived from gamma-glutamyl tyrosine (150). According to this study, the fat line’s gamma-glutamyl tyrosine concentration was much lower than the lean line’s, which may indicate that the fat birds’ ability to provide tyrosine was inferior to that of the lean line. According to Kabuki et al. (151), tyrosine had a physiological function that prevented stress. As a result, the lean birds may have high feed efficiency due to their excellent anti-stress capacity. Furthermore, as tyrosine is necessary for protein synthesis, the amount of gamma-glutamyl tyrosine may indicate the direction of an animal’s protein metabolism (30).

According to Zhou et al. (152), feed efficiency was enhanced by increased muscle yield. Additionally, Ramayo-Caldas et al. (153) discovered that feed efficiency decreased with increased belly fat accumulation. One vitamin D compound that is biologically active and works in several bodily tissues is calcitriol, which is created by the metabolism of vitamin D (154). Calcitriol guarantees the healthy growth and upkeep of bone and is vital for calcium and phosphate homeostasis (155). Furthermore, human studies have shown that thin people had far greater serum calcitriol levels than obese people (156). Six of the ten markers, epsilon-(gamma-glutamyl)-lysine, dimethyl sulfone, 2-oxoadipic acid, calcitriol, adenosine 5′-monophosphate, and 7-ketocholesterol, were discovered to be implicated in immunological response and reaction of inflammation (30). Genome-wide linkage research on the effectiveness of chicken feed claims that the immune system and inflammatory response may have an impact on feed efficiency (157). Both inflammatory responses and immunological responses may impact feed efficiency, according to transcriptome analyses of the chicken breast muscle (149), duodenum (158), jejunum (159), and liver (157). According to the biological roles of metabolites, the current study discovered that chickens with high feed efficiency exhibited increased immunity and decreased inflammation. Studies on inflammation and the immune response revealed that animals’ energy needs rose sharply during inflammation, which reduced the amount of energy available for protein deposition and, as a result, reduced feed efficiency (160).

The pathways and metabolites impacted by ellagic acid (EA) were investigated. According to metabolite data, EA influences muscle metabolism and nutritional value. EA controlled the concentrations of lipid metabolism-related metabolites taurine, hypotaurine, glycerophospholipid, and amino acids. Meat is evaluated for its nutritional value based on the levels of L-glutamine, L-proline, L-aspartic acid, and trans-4-hydroxy-L-proline (161). Glutamate-supplemented diets may specifically enhance meat quality by reducing water loss and glutamine metabolism, as well as making up for the rapid pH drop (162). The quantity of glycogen in muscle at slaughter has a negative correlation with the final pH of breast meat in broilers (163). Furthermore, one important component influencing the color of meat is the chemical condition of myoglobin (164). L-aspartic acid was found to have a positive correlation with intramuscular fat concentrations by Chen et al. (165), indicating that the quality of meat may be determined by this metabolite. In real-world applications, glutamine and aspartic acid help produce umami flavor (166). Taurine contributes to lipid homeostasis, stabilizes cell membranes, and possesses antioxidant properties (140). Lamb meat’s flavor and nutritional value are both improved by taurine (167). Furthermore, De Luca et al. (168) found that 0.5% taurine decreased glycolysis and the percentage of type-b muscle fibers in broiler thigh muscle, indicating a decrease in protein denaturation and an improvement in meat quality. Animals mostly manufacture proline from arginine (169). High meat nutritional value is associated with high proline concentrations (170). Meat’s collagen content has historically been estimated using the concentration of trans-4-hydroxy-l-proline (161).

Modern research by Wang et al. (140) showed that supplementing broilers with ellagic acid (EA) enhanced their growth performance, slaughter performance, organ indices, and meat quality. Additionally, EA additives changed the fatty acid and amino acid profiles of the muscles, indicating that EA increased the nutritional content of breast meat. The main metabolites found in breast meat were specifically L-aspartic acid, L-glutamine, taurine, L-proline, palmitoyl-L-carnitine, and trans-4-hydroxy-L-proline. According to the metabolic pathway study, purine and pyrimidine metabolism, primary bile acid biosynthesis, phenylalanine metabolism, and lysine metabolism were the differential metabolites linked to the enhancement of meat quality by CGA. Additionally, four metabolites—C5:1 acyl-carnitine, biotinamide, aminomalonic acid, and N-methyl-amino isobutyric acid—were found to be promising indicators for predicting broiler meat quality characteristics (171).

The quality of chicken meat, including characteristics like softness, color, pH, flavor, and water-holding capacity, is affected by a complex interaction of genetic, nutritional, and environmental factors. Metabolomics, which is the detailed study of small molecules in the body, is a powerful way to explore the biochemical reasons behind these characteristics, helping us understand how meat quality is determined (162). Metabolomic studies reveal unique metabolic patterns for different breeds, showing changes in amino acids (glutamate for umami) and nucleotides (inosine monophosphate for flavor) that relate to taste qualities (164, 165). The composition of feed, including omega-3 fatty acids and antioxidants, modifies lipid profiles and oxidative stability, hence affecting flavor and shelf life. Metabolites such as glutathione may function as indicators for resistance to oxidative stress (170).

Metabolomics offers a powerful tool for improving poultry growth and feed efficiency by identifying metabolites that serve as biomarkers for traits like feed intake, growth performance, and meat quality. It provides deeper insights into the biochemical pathways that affect poultry health and productivity. Key metabolites, such as epsilon-(gamma-glutamyl)-lysine and L-glutamine, are linked to inflammation and meat quality, highlighting the potential of metabolomics in breeding for better feed efficiency and meat characteristics. The use of additives like ellagic acid also demonstrates how nutrition can optimize growth and meat quality. Integrating metabolomic data into breeding and nutrition strategies could enhance poultry production efficiency and sustainability.

## 8 Applications of metabolomics for stress and welfare assessment

Commercial broilers with high metabolic rates and rapid growth are especially vulnerable to heat stress (172). The bird’s metabolic rate may rise under heat stress, which would result in an excess of reactive oxygen species. Heat stress can also interfere with mitochondrial activity, which can increase the generation of ROS (173). Heat stress-induced OS can affect a bird’s health in a variety of ways, such as producing too many ROS, causing lipid peroxidation, changing the structure and functionality of cell membranes, and oxidatively modifying nucleic acids and proteins (174). Furthermore, Shimamoto et al. (175) found that most plasma amino acid concentrations were not impacted by heat temperature, except for serine, glutamine, and tyrosine concentrations. This was especially noticeable in the poultry given the control diet, where the plasma glutamine concentration was considerably reduced by the hot temperature environment. This was in line with the results of another research that found that hens raised at high temperatures had lower levels of plasma glutamine along with worse carcass and performance characteristics (176). Despite being a non-essential amino acid, it has been demonstrated that glutamine regulates intestinal barrier function and encourages enterocyte survival and proliferation under a range of stressful situations (125). Additionally, it was discovered that heat-induced disruptions in intestinal shape and permeability were followed by a rise in the quantity of plasma endotoxins in broilers (177). These findings imply that under heat stress, broilers use glutamine to preserve intestinal integrity (175). They conducted an untargeted metabolomics study using GC–MS/MS to determine how the heat temperature conditions affected the profiles of metabolites in the chickens’ plasma. Of the 172 metabolites found in the plasma, 23 were found to be substantially impacted by the high temperature. The amounts of pyrimidine-related plasma (uracil, dihydrouracil, and thymine) and purine-related (hypoxanthine, inosine monophosphate, xanthosine, xanthine, and uric acid) metabolites were especially impacted by the high-temperature environment.

The enrichment analysis revealed that ammonia recycling, purine metabolism, pyrimidine metabolism, the urea cycle, homocysteine degradation, glutamate metabolism, *β*-alanine metabolism, glycine and serine metabolism, and aspartate metabolism were all altered by the high-temperature environment (175). Seven of these pathways (apart from aspartate metabolism and homocysteine degradation) support the results of a prior investigation on the impact of brief chronic heat on chicks’ plasma metabolomic profiles (178). It has been suggested that chickens raised at high temperatures have higher plasma homocysteine concentrations. In Japanese quail, heat temperature increased the homocysteine level and the lipid peroxidation concentration in the sample of plasma sample (179). Additionally, the hot temperature environment reduced the plasma concentration of aspartic acid, a precursor to produce pyrimidines, according to the untargeted GC–MS/MS-based metabolomics investigation. These findings imply that while the hot conditions in chickens increased the synthesis of purines and pyrimidines, they may have caused disruptions in the concentration of associated metabolites, such as aspartic, glutamine, and ascorbic acids (175). The birds given the baseline diet and housed in the heat had the lowest plasma level of *β*-alanine, although they had the highest plasma dihydrouracil content. Consequently, our findings imply that hens housed in hot temperatures may have problems converting dihydrouracil to β-alanine. A dose-dependent rise in the amount of carnosine in the brain and muscles was seen in birds given β-alanine orally (180). Thus, these findings imply that orotic acid preserves the antioxidative capacity of broilers under conditions of heat stress via changing the metabolism of *β*-alanine, which raises the muscle carnosine level.

Research shows orotic acid does not affect hen growth but reduces heat-induced lipid peroxidation levels in the plasma, according to Shimamoto et al. (175). Given that the plasma concentration of orotic acid decreased in hot weather and increased in response to orotic acid additives, this amelioration may have been caused in part by the antioxidative action of orotic acid (181). According to plasma analysis of metabolomics using GC–MS/MS, orotic acid additives impact twelve metabolites in plasma, including antioxidative uridine, 3-hydroxyanthranilic acid, tyrosine, glutamic acid, aspartic acid, and pyrimidines, according to GC–MS/MS analysis of plasma metabolomics (182). PRPP synthetase is crucial for purine and pyrimidine production, and supplementing with orotic acid significantly increases carnosine content and plasma β-alanine levels in chicken breast muscles (175).

Metabolomics plays a key role in assessing stress and welfare in poultry, particularly under heat stress conditions. Heat stress increases reactive oxygen species (ROS) production, leading to oxidative stress (OS), which can damage cell membranes, proteins, and nucleic acids. Studies show that heat stress reduces plasma glutamine levels and disrupts intestinal integrity, highlighting the importance of glutamine in maintaining gut health under stress. Metabolomic profiling has identified changes in metabolites, including purine and pyrimidine derivatives, amino acids, and metabolic pathways like ammonia recycling and glutamate metabolism, all of which are impacted by heat stress. Additionally, additives like orotic acid have been shown to reduce heat-induced lipid peroxidation and improve antioxidative capacity, potentially enhancing poultry welfare under heat stress.

## 9 Recent metabolomics in chicken meat quality

Given the rapidly expanding worldwide market, one of the most intensely targeted targets in modern metabolomics has been the quality traits of chicken meat (31). The 1H NMR-based method was used to study the meat of a Chinese indigenous breed of chicken called Wuding (183). The age of the chicken had an impact on the amounts of metabolites like lactate, IMP, glucose, creatine, carnosine, taurine, and anserine. Furthermore, a significant problem facing the poultry sector now is the unusual quality of chicken meat. A sharp increase in the size of the breast muscle has been linked to an increase in muscle abnormalities, such as wooden breast (WB), spaghetti meat (SM), and white striping (WS) phenotypes (184). Metabolomics techniques have been used to clarify the mechanisms behind the WB phenotype’s hardness. Abasht et al. (185) used GC–MS and LC–MS/MS, followed by RF method analysis, to identify chemicals linked to protein concentration, muscle protein breakdown, and changed glucose metabolism as potential biomarkers for WB.

According to a different study using a 1H–NMR technique, broiler breasts impacted by WB had lower levels of histidine, acetate, *β*-alanine, creatine, anserine, and creatinine than normal fillets, but higher concentrations of valine, leucine, alanine, lysine, lactate, glutamine, succinate, glucose, taurine, and IMP (186). Muscle drip was utilized as a sample in an additional effort to investigate WB myopathy biomarkers. Using an NMR method, nucleotides, amino acids, and organic acids were found to be the WB-associated metabolites linked to the samples’ ability to discriminate between WB and non-WB phenotypes (187), indicating the potential utility of those metabolites as WB markers. This outcome was somewhat in line with the earlier research that used breast samples that did or did not have WB (188). However, in a study using GC–MS and LC–MS techniques, WS was linked to altered metabolism of carbohydrates, long-chain fatty acids, and carnitine, indicating that altered *β*-oxidation plays a role in the WS phenotype (189). Low levels of anserine and carnosine are linked to pectoralis muscular dystrophy in chickens, according to research using an HR–MAS NMR technique (190). According to the findings of this metabolomics research, the anomalies in the chicken breast muscle may be due to changes in the metabolism of β-oxidation, amino acids and protein, and carbohydrates (31).

As a crucial component of phenomics, metabolomics is becoming a cutting-edge area of study in both medical and biological sciences. Numerous biological samples, such as extracts from various cells, organisms, tissues, and bodily fluids (such as urine, serum/plasma, exhaled air, tears, saliva, CSF, synovial fluid, and sputum), have been used to measure metabolomic differences. There are two pathways of metabolomics: targeted metabolomics and untargeted metabolomics. Using selected/multiple reaction monitoring (SRM/MRM) techniques, the known metabolite profile is analyzed using targeted metabolomics. With NMR-based techniques (1D-2D-NMR, NMR, and 3D-NMR) and MS-based techniques (LC–MS, DI-MS, GC–MS, IM-MS, and CE-MS), untargeted metabolomics is utilized to examine the unknown metabolite profile broadly (191).

Recent metabolomics research has focused on improving chicken meat quality, especially addressing muscle abnormalities like wooden breast (WB) and white striping (WS). Studies show that chicken age affects metabolites like lactate and taurine, which influence meat quality. WB is linked to changes in protein breakdown and glucose metabolism, while WS is associated with altered fatty acid metabolism. Metabolomics techniques, such as GC–MS and LC–MS/MS, help identify biomarkers for these conditions. Both targeted and untargeted approaches are used to analyze metabolites, offering insights into the biochemical factors affecting meat quality.

## 10 Future perspectives in poultry nutrition and metabolomics

Since both precision livestock farming and precision nutrition aim to increase farm profitability, efficiency, and sustainability, precision nutrition is a crucial component of precision animal farming (192). Biotechnological, metabolomic, computational, and protein engineering are some of the possible technologies for creating future precision feeding plans that will enhance broiler amino acid consumption (4). There are many applications of metabolomics in different fields. In the next section, focus on new applications of metabolomics.

### 10.1 Single-cell metabolomics

Most researchers agree that cells are the smallest, most fundamental building blocks of life. Cells in biological systems differentiate according to their genetic expression, forming diverse populations that can subsequently interact and form intricate structures such as tissues (193). Because biological functions might differ significantly among cells, bulk assessments of pooled cells are unable to distinguish between the diverse functions of different populations. Since single-cell omics analyses can identify cellular heterogeneity within tissues, they have grown in popularity to preserve this information (194). Individual cell-specific compounds can be extracted and measured using single-cell omics. These biomolecules can thereafter be contrasted with other cells to uncover distinct populations that are not detectable in bulk analysis (195). To demonstrate the functional connection between single-cell genotype and molecular phenotype, there has been an increase in single-cell metabolomics and proteomics data, even if single-cell transcriptomics has developed the fastest of all single-cell omics technologies (196). The most effective method for biomolecular profiling in single-cell metabolomics is probe-based MS, sometimes referred to as MS imaging. By employing probes, like an ion beam or laser, to carry out chemical absorption and ionization, mass spectrometry imaging (MSI) can determine the concentrations and locations of biomolecules (193, 197). Mass spectra can be attributed to cells in tissue by superimposing MSI probe ablation coordinates with cell pictures from the same sample slide (198). This avoids the need for single-cell isolation, which is an expensive and time-consuming procedure that is crucial to single-cell transcriptomic techniques (199). In biological MSI, precise spatial resolution is vital due to the small size of bacteria (1–2 μm) and eukaryotic (10–20 μm) cells (193). Single-cell analysis faces challenges as metabolites exist at minute abundances, differing from bulk analyses. Ion competition during ionization further limits detection, favoring only the most abundant metabolites. This necessitates optimized probe settings to enhance sensitivity and specificity. Achieving reliable metabolite profiling thus depends on balancing spatial precision and analytical sensitivity (200).

### 10.2 Volatile organic compounds (VOCs) feed

A new revolution in healthcare is being brought about by the development of metabolomic-based technology. Metabolites whose abundances are suggestive of phenotypic alterations have been identified by recent research. For instance, volatile organic compounds (VOCs) are one class of substances being studied as diagnostic markers. Sensors designed to swiftly monitor distinct metabolites have emerged in tandem with this trend. According to Soni et al. (200), the combination of these developments could result in tailored dietary advice, early disease detection, feed desirability, and many other uses. In GI tract tissues impacted by disease, changes to normal metabolic processes and physiology result in the production of VOCs (201). Noninvasive methods are used to quantify VOCs, which may be important components in the early diagnosis of numerous illnesses. Electronic nose (e-nose) instruments are devices that use a variety of sensor arrays to test a wide range of volatile organic compounds. Colorectal cancer, ulcerative colitis, Crohn’s disease, cholera, and irritable bowel syndrome are among the GI tract conditions that can be identified by e-noses (201).

### 10.3 Artificial intelligence (ML and DL)

Applications for machine learning (ML) and deep learning (DL) are used in many aspects of daily life, such as chatbots for customer support, spam filtering, product suggestions, and language translation. The use of omics data in the artificial intelligence revolution has garnered significant attention in the healthcare industry, as seen by some research studies (202–204). However, in contrast to other omics, like transcriptomics and genomics, which have access to numerous validated datasets and tools, metabolomics analytic pipelines are far less developed (205, 206). Researchers are coming up with several ways to improve the robustness of metabolomic annotations by utilizing ML and DL. To identify tiny chemicals using electron ionization mass spectrometry (EI-MS) with limited resolution, Pomyen et al. (207) developed a deep convolutional neural network (CNN).

## 11 Conclusion

Accurately addressing the nutritional requirements of animals for safe, superior, and effective production is known as precision feeding. This multidisciplinary approach integrates traditional nutrition with various fields like biology, computer science, immunology, genetics, molecular biology, chemistry, biochemistry, mathematics, engineering, and technology sciences. With a molecular weight of fewer than 1,500 Daltons, smaller than most proteins, DNA, and other cellular macromolecules, metabolomics is a growingly popular technique that sets itself apart from other “omics” topics. There are numerous metabolomics techniques, including emerging capillary electrophoresis (CE), gas chromatography mass spectrometry (GC–MS), nuclear magnetic resonance spectroscopy (NMR), liquid chromatography mass spectrometry (LC–MS), and several targeted HPLC-based assays. Inductively coupled plasma mass spectrometry (ICP-MS) is another technique. Numerous factors, including protein production rate, consumption and metabolic rate, and protein loss, influence serum total protein. A vital component of the system of enzymes, zinc, also participates in the production of proteins, the metabolism of carbohydrates, and several other biochemical processes. Numerous metabolites might be employed as potential biomarkers for poultry growth and efficiency, including dimethyl sulfone, 7-ketocholesterol, epsilon-(gamma-glutamyl)-lysine, 2-oxoadipic acid, gamma-glutamyltyrosine, L-homoarginine, adenosine 5′-monophosphate, testosterone, calcitriol, and adrenic acid. Supplementation with precision nutrition increases growth performance, meat quality, slaughter performance, and organ indices of chickens. Future approaches in poultry nutrition and metabolomics, like artificial intelligence, Machine learning, deep learning, and single-cell metabolomics applications. Metabolomics enables precise feed optimization by identifying biomarkers linked to poultry health and growth, reducing feed costs (10–15%) via targeted nutrient formulations. Early disease detection through metabolic profiles cuts treatment expenses and mortality (5–7%). Enhanced gut health insights improve FCR by 5–10%, while metabolite-driven breeding boosts resilience. Initial setup costs are offset by long-term savings in feed, health, and productivity, with sustainability gains from reduced waste and emissions. Prioritize partnerships with labs for scalable, cost-effective implementation. In conclusion, the Integrating Metabolomics and Precision Nutrition in Poultry had positive effects on performance, immunity, antioxidant, heat stress, and discovered new biomarkers (metabolomes) for these important traits.
